# Associations between frequency of yogurt consumption and nutrient intake and diet quality in the United Kingdom

**DOI:** 10.1017/jns.2021.63

**Published:** 2021-10-04

**Authors:** Yong Zhu, Neha Jain, Norton Holschuh, Jessica Smith

**Affiliations:** 1 Bell Institute of Health and Nutrition, General Mills, Inc., Minneapolis, MN 55427, USA; 2 Global Knowledge Solutions, General Mills Canada Corporation, Mississauga, ON L4W 5N9, Canada; 3 Global Knowledge Solutions, General Mills, Inc., Minneapolis, MN 55427, USA

**Keywords:** Diet quality, Dietary intake, National Diet and Nutrition Survey, Yogurt

## Abstract

Little is known on the association between frequency of yogurt consumption and dietary intake in the United Kingdom (UK). The aim of the present study was to examine associations between frequency of yogurt consumption and dietary outcomes in children (*n* 1912, age 9⋅6 ± 0⋅1 years, 51 % boys) and adults (*n* 2064, age 48⋅7 ± 0⋅5 years, 49 % men) using cross-sectional data from the National Diet and Nutrition Survey rolling programme year 7 to year 9 (2014/15–2016/17). The frequency of yogurt consumption was determined by the number of days with yogurt reported in 4-d food diaries and participants were classified as non-eaters, occasional eaters (1–2 d of consumption) or regular eaters (3–4 d of consumption). Dietary outcomes were estimated from food diaries. The frequency of yogurt consumption was positively associated with intake of key vitamins and minerals such as thiamin, riboflavin, vitamin C, potassium, calcium, magnesium, phosphorus and iodine in both children and adults (all *P* < 0⋅0018), as well as higher intake of total dairy (*P* < 0⋅0001 for both children and adults). Regular yogurt eaters were more likely to meet or exceed nutrient recommendations for vitamins and minerals such as vitamin A, riboflavin, folate, potassium, calcium, magnesium, zinc and iodine (all *P* < 0⋅001). Diet quality was positively associated with frequency of yogurt consumption in children (*P* = 0⋅045) and adults (*P* < 0⋅001). No association between yogurt consumption and free sugar intake was found (*P* = 0⋅49 for children and *P* = 0⋅29 for adults). The study suggests that frequency of yogurt consumption is associated with better dietary intake and diet quality in children and adults in the UK.

## Introduction

Yogurt, a nutrient-dense fermented dairy product, has been recommended in many food-based dietary guidelines^(1)^, including the United Kingdom (UK) Eatwell Guide^(2)^, as part of a healthy balanced diet. Numerous review articles have summarised the association between yogurt consumption and health benefits such as weight management^(3,4)^, healthy aging^(5)^, cardiometabolic health^(6)^, gut health^(7)^, bone health^(8)^ and skin health^(9)^. The unique health benefit of yogurt consumption is likely to be mediated by the nature of yogurt matrix due to fermentation by bacteria and its nutrient composition. For example, bioactive fatty acids, whey and casein, calcium, as well as lactic acid bacteria, are proposed to be responsible for mechanisms of action for the beneficial effect of yogurt on cardiometabolic health^(10)^.

Review of epidemiological studies revealed that yogurt consumers had a healthier diet and a healthier lifestyle than non-consumers^(11)^, which could further account for positive associations between yogurt consumption and health outcomes. Studies of either nationally representative samples or local populations consistently reported that yogurt consumption was associated with better nutrient intake or improved diet quality in different countries, such as United States^(12–14)^, Canada^(15)^, Italy^(16)^ and Spain^(17)^, despite possible differences in food culture in these countries. Similar results were reported in the United Kingdom using earlier data (2008/9 to 2010/11 or 2008/9 to 2011/12) from the National Diet and Nutrition Survey (NDNS), showing that yogurt made a meaningful contribution to nutrient intake across the life course in the British population^(18)^, and a higher amount of yogurt consumed was positively associated with higher nutrient intake such as calcium, iodine and riboflavin, as well as improved dietary quality in British children^(19)^. However, it is not known if there was any change in yogurt consumption pattern in recent years, and whether recent yogurt product reformulation, such as sugar reduction, changes in fortification or shifts in milkfat content^(20,21)^ had changed associations between yogurt intake and nutrient intake and diet quality.

Therefore, the objectives of the present study were to examine associations between frequency of yogurt consumption and nutrient intake, nutrient adequacy, and diet quality using the most recent data from the NDNS (2014/15 to 2016/17), and to assess contribution of yogurt to nutrient intake, in both British children and adults. In addition, the study sought to investigate relationships between yogurt consumptions and intake of food groups that were recommended in the UK Eatwell Guide, which had not been studied previously, to the best of our knowledge.

## Methods

### Design and study population

The NDNS rolling programme is a continuous cross-sectional survey funded by Public Health England and the UK Food Standards Agency for assessment of dietary intake and nutrition status in the UK since 2008^(22)^. Each year, surveys are conducted in a nationally representative sample for participants aged 1⋅5 years or older and collect information on demographic characteristics, detailed dietary intake, and health status and history; the survey data are publicly available^(23)^. Detailed survey information is described elsewhere^(24)^. The present study included participants from year 7 to year 9 of the NDNS (2014/15 to 2016/17) whose interview status was considered to be fully productive as determined by the NDNS (*n* 3976). The present study did not include pregnant or breastfeeding women as those participants were assigned to another category for their interview status in the NDNS data. The NDNS was conducted according to the guidelines from the Declaration of Helsinki and ethics approval for year 7 to year 9 of the NDNS was obtained from the Cambridge South NRES Committee; all participants (or their parents or guardians for children aged under 16 years) provided their informed consent^(22)^.

Details for dietary data collection and processing were provided elsewhere^(25)^. Briefly, four consecutive days of food diaries were collected by trained interviewers from the study team who visited each participant three times. Depending on the day of the first diary, the four days of food diaries might include both weekdays and weekend days (72 % of children and 72 % of adults included in this study had at least one food diary from weekend). During the first visit, instruction was provided, and blank diaries were provided. Participants or their proxies (for children aged 11 years or younger) were asked to record everything eaten and drunk. Participants were asked to take the diary with them when they were away from home, and for young children, adults who were with the children might have completed parts of the diary. For packaged foods, brand names were recorded wherever possible; for homemade dishes, individual ingredients, quantities and cooking method were asked to be recorded on a separate sheet. For portion size, the proxies for children were asked to report portion size in household measures or weights from food packages; for adults, they also received photographs of ten common foods in small, medium and large portions to help with portion size estimate. During the diary intake collection period, a second visit was made for compliance checking, answering question, reviewing diary data, editing possible omissions or adding details missed. The last visit occurred within 3 d of the last day of the diary to review and collect the diaries. For dietary data processing, diaries were entered into a modified version of the Medical Research Council Elsie Widdowson Laboratory's Dietary Assessment System with built-in data from the Public Health England's NDNS Nutrient Databank, to convert food data into nutrient data^(25)^.

### Exposure assessment

The NDNS dietary data classified foods into main food groups; yogurt, fromage frais and dairy desserts is one of the main good groups. Similar to a previous NDNS study^(19)^, in the present study, yogurt was defined as all types of yogurt and fromage frais and excluded dairy desserts. Dairy desserts were excluded due to differences in dietary use, eating occasions and nutrition profiles compared with yogurt and fromage frais. Participants were classified by the number of days with reported consumption of yogurt in their 4-d food diaries: regular consumers (at least 3 d), occasional consumers (1 or 2 d) or non-consumers (0 d).

### Outcome variables

Energy and nutrient intake were estimated as average daily intake from the 4-d food diaries and did not include intake from dietary supplements, similar to analysis of nutrient intake presented in the official NDNS year 1 to year 9 report^(24)^. The list of nutrients included was chosen based on those presented in an earlier study^(19)^ for comparisons. Food groups examined were chosen based on those food groups referenced in the UK Eatwell Guide and included fruit and fruit juice, vegetables, dairy and dairy alternatives (those from the ‘other milk’ or ‘other cheese’ food groups in NDNS, such as soy milk, almond milk, oat-based milk, tofu cheese), starchy carbohydrates, protein foods, oils, spread and solid fat, and mixed dishes; they were estimated as average daily intake after combining relevant food groups in the NDNS data. For yogurt consumers, percent contribution to daily energy and nutrient intake from yogurt was estimated using average daily intake from days when yogurt was consumed. Nutrient adequacy for vitamins and minerals was assessed as percentages of participants whose usual intake (estimated as average intake from 4-d food diaries) were below lower reference nutrient intakes (LRNI) in the UK^(26)^ for nutrients with established LRNI, which is determined as two standard deviations below the Estimated Average Requirements, and any intake below this level are ‘almost certainly inadequate for most individuals’^(26)^.

Similar to a previous study that used NDNS dietary data^(27)^, diet quality was assessed by the Nutrient Rich Food Index 9.3 (NRF 9.3), a validated nutrient profiling model that was adapted to be used for assessment of quality of total dietary intake^(28–30)^. Nutrients included in the NRF 9.3 were nine nutrients to encourage (protein, fibre, vitamin A, vitamin C, vitamin D, calcium, iron, potassium, magnesium) and three nutrients to limit (saturated fat, salt and total sugar); daily intake for each nutrient in the model was standardised to 2000 kcal and compared with reference intakes from EU Nutrient Reference Values (EU Regulation No 1169/2011)^(31)^ to get a subscore for that nutrient, and the total score was calculated as the sum of subscores for nutrients to encourage minus the sum of subscores for nutrients to limit. The reference intakes were 50 g for protein, 30 g for fibre, 800 μg for vitamin A, 5 μg for vitamin D, 80 mg for vitamin C, 2000 mg for potassium, 800 mg for calcium, 375 mg for magnesium, 14 mg for iron, 20 g for saturated fat, 90 g for sugar and 6 g for salt, all standardised to a 2000 kcal diet.

### Covariates

Age, gender and equivalised household income tertiles were included as covariates for analysis of energy intake and diet quality. Analysis of nutrient intake and intake of food groups were adjusted for the same covariates and energy intake. Age and energy intake were used as continuous variables, whereas gender and equivalised household income tertiles were used as categorical variables. Missing information for a given covariate was coded as a separate category^(32–34)^.

### Statistical methods

SAS 9.4 (SAS Institute, Cary, NC, USA) was used for statistical analysis. Survey weights were applied in models to account for the multi-stage survey design in NDNS. Power analysis revealed that 174 subjects per group would be required for an effect size of 0⋅3 with 80 % power at the significance level of 0⋅05^(35)^. The characteristics of participants by frequency of yogurt consumption were compared by ANOVA for continuous variables or *χ*^2^ test for categorical variables. Differences in dietary intake and diet quality by yogurt consumption status were compared using multiple linear regression analyses, followed by pair-wise comparisons. Data for children aged 1⋅5–18 years (*n* 1912) and adults 19 years or older (*n* 2064) were analysed separately. Data were presented as weighted percentages or mean with their standard error. For each set of outcomes, Bonferroni-corrected *P* values were used to determine significant associations to account for multiple comparisons, followed by Tukey *post hoc* comparisons within three groups by frequency of yogurt consumption.

## Results

### Frequency of yogurt consumption and participants’ characteristics

Children (51 % boys) had a mean age of 9⋅6 ± 0⋅1 years and adults (49 % men) had a mean age of 48⋅7 ± 0⋅5 years. Table 1 presents frequency of yogurt consumption in children and adults as well as its association with age, gender and equivalised household income tertiles. Fifty-two percent of children in the UK were yogurt consumers with 36 % being occasional eaters and 16 % being regular eaters. By contrast, 25 % of adults were occasional eaters and 14 % of adults were regular eaters in the UK. The total amount of yogurt consumed over 4 d in regular eaters and occasional eaters was 343⋅4 and 143⋅5 g, respectively, in children, and 488⋅6 and 173⋅7 g, respectively, in adults; whereas the average portion size (amount consumed in a single eating occasion) in regular eaters and occasional eaters was 83⋅4 and 95⋅7 g, respectively, in children, and 115⋅3 and 117⋅0 g, respectively, in adults (data not shown). There was an inverse relationship between age and frequency of yogurt consumption in children (*P* < 0⋅001): the mean age of children who were regular eaters was significantly younger than non-eaters and occasional eaters. For adults, a positive relationship between age and frequency of yogurt consumption was found (*P* < 0⋅001): those who were regular eaters were older than adults who were non-eaters or occasional eaters. There was no association between gender and yogurt consumption in children (*P* = 0⋅95), however, women were more likely to be yogurt consumers than men (*P* < 0⋅001). Equivalised household income tertiles were significantly associated with frequency of yogurt consumption in both children (*P* = 0⋅005) and adults (*P* = 0⋅02); those who were from the highest tertile in equivalised household income were more likely to be yogurt consumers.

**Table 1.** Characteristics of participants by frequency of yogurt consumption in the United Kingdom, National Diet and Nutrition Survey 2014/15 to 2016/17*

	Children 1⋅5–18 years							Adults 19 years or older						
	Non-eaters *n* 868 (48 %)		Occasional eaters *n* 717 (36 %)		Regular eaters *n* 327 (16 %)			Non-eaters *n* 1264 (62 %)		Occasional eaters *n* 526 (25 %)		Regular eaters *n* 274 (14 %)		
	%	se	%	se	%	se	*P*	%	se	%	se	%	se	*P*
Age (years)							<0⋅001							<0⋅001
Mean	11⋅5		8⋅2		6⋅8			47⋅7		47⋅9		54⋅5		
se	0⋅2		0⋅2		0⋅3			0⋅7		1⋅0		1⋅3		
Gender							0⋅95							<0⋅001
Male	51⋅8	2⋅1	50⋅7	2⋅4	51⋅1	3⋅4		54⋅3	1⋅7	40⋅0	2⋅7	38⋅9	3⋅6	
Female	48⋅2	2⋅1	49⋅3	2⋅4	48⋅9	3⋅4		45⋅7	1⋅7	60⋅0	2⋅7	61⋅1	3⋅6	
Equivalised household income tertiles							0⋅005							0⋅02
Lowest tertile	38⋅3	2⋅1	35⋅4	2⋅3	29⋅7	3⋅2		25⋅0	1⋅5	22⋅8	2⋅3	19⋅1	2⋅8	
Middle tertile	24⋅9	1⋅8	22⋅9	1⋅9	26⋅8	3⋅0		24⋅8	1⋅5	23⋅4	2⋅3	22⋅2	3⋅0	
Highest tertile	21⋅6	1⋅6	28⋅2	2⋅0	34⋅3	3⋅2		32⋅4	1⋅7	38⋅9	2⋅6	45⋅7	3⋅6	
Missing	15⋅3	1⋅7	13⋅4	1⋅7	9⋅1	2⋅1		17⋅7	1⋅4	14⋅9	2⋅1	12⋅9	2⋅3	

* Data were compared by ANOVA for continuous variables or *χ*^2^ test for categorical variables.

### Energy and nutrient intake

Associations between frequency of yogurt consumption and energy and nutrient intake are presented in Table 2. In both children and adults, frequent yogurt consumption was positively associated with energy intake (both *P* < 0⋅001). In children, yogurt consumption was also positively associated with intake of total sugar and several vitamins and minerals such as thiamin, riboflavin, vitamin C, vitamin D, potassium, calcium, magnesium, phosphorus and iodine (all *P* ≤ 0⋅0018). In adults, yogurt consumption was positively associated with intake of total sugar, fibre (reported as AOAC fibre in year 7 to year 9 of the NDNS dietary data), thiamin, riboflavin, folate, vitamin C, potassium, calcium, magnesium, phosphorus, zinc, iodine and manganese (all *P* ≤ 0⋅0018).

**Table 2.** Adjusted mean energy and nutrient intake by frequency of yogurt consumption in children and adults in the United Kingdom, National Diet and Nutrition Survey 2014/15 to 2016/17*

	Children 1⋅5–18 years							Adults 19 years or older						
	Non-eaters *n* 868 (48 %)		Occasional eaters *n* 717 (36 %)		Regular eaters *n* 327 (16 %)			Non-eaters *n* 1264 (62 %)		Occasional eaters *n* 526 (25 %)		Regular eaters *n* 274 (14 %)		
	Mean	se	Mean	se	Mean	se	*P*	Mean	se	Mean	se	Mean	se	*P*
Energy (kcal)	1436⋅2^a^	18⋅0	1467⋅8^a^	18⋅9	1559⋅6^b^	25⋅3	0⋅0002	1720⋅5^a^	20⋅0	1899⋅8^b^	27⋅8	1876⋅9^b^	36⋅5	<0⋅0001
Protein (g)	55⋅7^a^	0⋅5	56⋅0^a^	0⋅5	57⋅5^a^	0⋅7	0⋅09	71⋅4^a^	0⋅6	74⋅1^ab^	1⋅3	76⋅4^a^	1⋅4	0⋅002
Fat (g)	56⋅3^a^	0⋅4	55⋅4^ab^	0⋅4	54⋅0^b^	0⋅5	0⋅003	66⋅1^a^	0⋅5	66⋅2^a^	0⋅7	63⋅8^a^	1⋅1	0⋅12
Saturated fat (g)	21⋅5^a^	0⋅2	21⋅2^a^	0⋅2	21⋅3^a^	0⋅3	0⋅77	24⋅4^a^	0⋅3	24⋅8^a^	0⋅4	23⋅4^a^	0⋅6	0⋅11
Carbohydrate (g)	197⋅8^a^	1⋅0	199⋅7^a^	1⋅0	201⋅4^a^	1⋅5	0⋅17	210⋅7^a^	1⋅4	210⋅5^a^	2⋅4	219⋅2^b^	2⋅8	0⋅02
Total sugar (g)	79⋅4^a^	1⋅1	85⋅3^b^	1⋅0	90⋅0^c^	1⋅6	<0⋅0001	85⋅9^a^	1⋅2	89⋅6^a^	1⋅7	100⋅8^b^	2⋅4	<0⋅0001
Free sugar (g)	51⋅6^a^	1⋅0	52⋅8^a^	1⋅0	53⋅7^a^	1⋅5	0⋅49	53⋅0^a^	1⋅3	50⋅5^a^	1⋅7	49⋅6^a^	2⋅0	0⋅29
AOAC Fibre (g)	13⋅7^a^	0⋅2	14⋅2^b^	0⋅2	14⋅1^ab^	0⋅2	0⋅04	17⋅8^a^	0⋅3	18⋅8^a^	0⋅3	21⋅4^b^	0⋅7	<0⋅0001
Vitamin A (μg)	491⋅6^a^	13⋅4	514⋅6^ab^	14⋅5	567⋅2^b^	23⋅4	0⋅03	869⋅6^a^	42⋅2	936⋅7^a^	47⋅2	908⋅0^a^	54⋅8	0⋅49
Thiamin (mg)	1⋅2^a^	0⋅0	1⋅3^b^	0⋅0	1⋅3^b^	0⋅0	0⋅0002	1⋅4^a^	0⋅0	1⋅5^a^	0⋅0	1⋅7^b^	0⋅1	<0⋅0001
Riboflavin (mg)	1⋅3^a^	0⋅0	1⋅4^b^	0⋅0	1⋅5^c^	0⋅0	<0⋅0001	1⋅5^a^	0⋅0	1⋅6^b^	0⋅0	1⋅8^c^	0⋅0	<0⋅0001
Niacin (mg)	25⋅9^a^	0⋅3	25⋅8^a^	0⋅3	26⋅0^a^	0⋅4	0⋅87	34⋅1^a^	0⋅4	33⋅8^a^	0⋅6	35⋅2^a^	0⋅8	0⋅29
Vitamin B6 (mg)	1⋅3^a^	0⋅0	1⋅4^a^	0⋅0	1⋅4^a^	0⋅0	0⋅06	1⋅7^ab^	0⋅0	1⋅6^b^	0⋅0	1⋅8^a^	0⋅0	0⋅01
Folate (μg)	166⋅3^a^	2⋅6	175⋅5^b^	2⋅8	179⋅4^b^	3⋅7	0⋅01	227⋅1^a^	3⋅0	232⋅8^a^	4⋅5	264⋅2^b^	8⋅9	0⋅0005
Vitamin B12 (μg)	3⋅8^a^	0⋅1	3⋅9^a^	0⋅1	4⋅1^a^	0⋅1	0⋅21	4⋅9^a^	0⋅1	5⋅1^a^	0⋅1	5⋅4^a^	0⋅2	0⋅104
Vitamin C (mg)	69⋅4^a^	1⋅9	79⋅9^b^	2⋅2	79⋅1^b^	3⋅0	0⋅0007	75⋅1^a^	1⋅9	83⋅9^b^	3⋅0	100⋅4^c^	6⋅1	0⋅0002
Vitamin D (μg)	1⋅9^a^	0⋅1	2⋅0^a^	0⋅1	2⋅5^b^	0⋅1	<0⋅0001	2⋅6^a^	0⋅1	2⋅8^a^	0⋅1	3⋅5^b^	0⋅2	0⋅002
Vitamin E (mg)	7⋅7^a^	0⋅1	7⋅6^a^	0⋅1	7⋅6^a^	0⋅1	0⋅66	9⋅2^a^	0⋅1	9⋅5^ab^	0⋅2	10⋅2^b^	0⋅3	0⋅01
Sodium (mg)	1637⋅0^a^	18⋅0	1606⋅9^a^	17⋅6	1575⋅9^a^	22⋅2	0⋅09	1996⋅5^a^	21⋅2	1951⋅3^ab^	30⋅4	1864⋅2^b^	38⋅2	0⋅01
Potassium (mg)	1999⋅3^a^	16⋅4	2096⋅7^b^	18⋅9	2169⋅9^b^	27⋅1	<0⋅0001	2684⋅5^a^	21⋅3	2792⋅3^b^	32⋅9	3119⋅8^c^	52⋅4	<0⋅0001
Calcium (mg)	704⋅1^a^	10⋅1	742⋅3^b^	11⋅5	826⋅2^c^	18⋅1	<0⋅0001	754⋅3^a^	9⋅1	818⋅4^b^	12⋅8	927⋅8^c^	21⋅3	<0⋅0001
Magnesium (mg)	182⋅6^a^	1⋅5	191⋅1^b^	1⋅6	194⋅0^b^	2⋅2	<0⋅0001	249⋅0^a^	2⋅5	261⋅0^b^	3⋅7	283⋅6^c^	5⋅1	<0⋅0001
Phosphorus (mg)	931⋅3^a^	7⋅7	964⋅6^b^	8⋅0	1022⋅6^c^	12⋅3	<0⋅0001	1157⋅6^a^	8⋅4	1223⋅6^b^	14⋅0	1329⋅7^c^	19⋅7	<0⋅0001
Iron (mg)	7⋅9^a^	0⋅1	8⋅1^a^	0⋅1	8⋅0^a^	0⋅1	0⋅13	9⋅8^a^	0⋅1	10⋅2^ab^	0⋅2	10⋅7^b^	0⋅3	0⋅01
Zinc (mg)	6⋅2^a^	0⋅1	6⋅3^ab^	0⋅1	6⋅4^b^	0⋅1	0⋅04	8⋅1^a^	0⋅1	8⋅5^b^	0⋅1	8⋅6^b^	0⋅1	0⋅0013
Iodine (μg)	113⋅4^a^	2⋅7	124⋅3^b^	2⋅6	139⋅3^c^	4⋅4	<0⋅0001	145⋅3^a^	2⋅3	165⋅2^b^	5⋅6	179⋅3^b^	6⋅4	<0⋅0001
Selenium (μg)	33⋅5^a^	0⋅6	33⋅3^a^	0⋅4	35⋅1^a^	0⋅8	0⋅12	46⋅1^a^	0⋅6	48⋅3^ab^	1⋅2	50⋅6^b^	1⋅6	0⋅02
Manganese (mg)	2⋅1^a^	0⋅0	2⋅2^a^	0⋅0	2⋅1^a^	0⋅0	0⋅12	3⋅0^a^	0⋅0	3⋅1^a^	0⋅1	3⋅5^b^	0⋅1	0⋅0009

* *P* values ≤0⋅0018 were considered to be statistically significant to account for comparisons of twenty-eight outcomes. Different letters in the same row within the data panel for children or adults indicate a significant difference from Tukey's *post hoc* comparison. Data were analysed by multiple linear regression analysis for survey data. Energy intake was adjusted for age, gender and equivalised household income tertiles, and nutrient intake was for age, gender, equivalised household income tertiles and energy intake.

### Food group intake

Intake of food groups by frequency of yogurt consumption is presented in Table 3. In children, frequency of yogurt consumption was positively associated with intake of dairy and dairy alternatives (*P* < 0⋅0001), and it was negatively associated with intake of starchy carbohydrates (*P* = 0⋅0012). For adults, frequency of yogurt consumption was positively associated with intake of fruit and fruit juice (*P* = 0⋅0007), vegetables (*P* < 0⋅0001) and dairy and dairy alternatives (*P* < 0⋅0001).

**Table 3.** Adjusted mean intake of food groups by frequency of yogurt consumption in children and adults in the United Kingdom, National Diet and Nutrition Survey 2014/15 to 2016/17*

	Children 1⋅5–18 years							Adults 19 years or older						
	Non-eaters *n* 868 (48 %)		Occasional eaters *n* 717 (36 %)		Regular eaters *n* 327 (16 %)			Non-eaters *n* 1264 (62 %)		Occasional eaters *n* 526 (25 %)		Regular eaters *n* 274 (14 %)		
	Mean	se	Mean	se	Mean	se	*P*	Mean	se	Mean	se	Mean	se	*P*
Fruits and fruit juice (g)	177⋅4^a^	7⋅0	191⋅2^a^	6⋅4	203⋅3^a^	11⋅0	0⋅10	162⋅1^a^	5⋅9	177⋅3^a^	17⋅1	231⋅5^b^	6⋅9	0⋅0007
Vegetables (g)	80⋅1^a^	2⋅7	89⋅5^a^	2⋅9	89⋅5^a^	3⋅6	0⋅04	161⋅6^a^	3⋅8	181⋅6^b^	9⋅5	207⋅8^c^	6⋅3	<0⋅0001
Dairy and dairy alternatives (g)	292⋅7^a^	9⋅1	391⋅9^b^	10⋅7	403⋅4^b^	14⋅2	<0⋅0001	236⋅1^a^	7⋅1	372⋅4^b^	13⋅2	384⋅1^b^	11⋅5	<0⋅0001
Starchy carbohydrates (g)	223⋅3^a^	3⋅1	214⋅1^ab^	3⋅3	203⋅6^b^	4⋅3	0⋅0012	260⋅2^a^	3⋅5	250⋅6^a^	5⋅9	244⋅4^a^	5⋅0	0⋅05
Protein foods (g)	124⋅4^a^	2⋅7	120⋅5^a^	2⋅3	117⋅4^a^	3⋅7	0⋅26	186⋅5^a^	3⋅0	187⋅2^a^	6⋅1	181⋅6^a^	5⋅2	0⋅73
Oil, spread and solid fat (g)	12⋅1^a^	0⋅4	11⋅9^a^	0⋅4	10⋅9^a^	0⋅5	0⋅11	17⋅2^ab^	0⋅3	17⋅5^a^	0⋅7	15⋅4^b^	0⋅5	0⋅04
Mixed dishes (g)	121⋅2^a^	4⋅7	111⋅9^ab^	4⋅4	100⋅2^b^	5⋅8	0⋅01	146⋅1^a^	4⋅6	129⋅5^ab^	9⋅6	116⋅6^b^	7⋅3	0⋅01

* *P* values ≤0⋅007 were considered to be statistically significant to account for comparisons of seven outcomes. Different letters in the same row within the data panel for children or adults indicate a significant difference from Tukey's *post hoc* comparison. Data were analysed by multiple linear regression analysis for survey data, adjusting for age, gender, equivalised household income tertiles and energy intake.

### Nutrient adequacy

The percentages of children and adults below LRNI for vitamin A, thiamin, riboflavin, niacin, vitamin B6, folate, vitamin B12, vitamin C, potassium, calcium, magnesium, iron, zinc, iodine and selenium are presented in Table 4. Frequency of yogurt consumption was positively associated with meeting or exceeding recommendations for vitamin A, riboflavin, folate, potassium, calcium, magnesium, zinc and iodine in both children and adults (all *P* < 0⋅0001). Moreover, frequency of yogurt consumption was positively associated with meeting or exceeding recommendations for iron and selenium in children (both *P* < 0⋅0001) and for vitamin C in adults (*P* < 0⋅0001). Occasional and regular yogurt eaters generally were less likely to have intakes below recommendations than non-eaters.

**Table 4.** Percentage of population below lower reference nutrient intake by frequency of yogurt consumption in children and adults in the United Kingdom, National Diet and Nutrition Survey 2014/15 to 2016/17*

	Children 1⋅5–18 years							Adults 19 years or older						
	Non-eaters *n* 868 (48 %)		Occasional eaters *n* 717 (36 %)		Regular eaters *n* 327 (16 %)			Non-eaters *n* 1264 (62 %)		Occasional eaters *n* 526 (25 %)		Regular eaters *n* 274 (14 %)		
	%	se	%	se	%	se	*P*	%	se	%	se	%	se	*P*
Vitamin A	19⋅0^a^	1⋅7	15⋅4^a^	1⋅9	6⋅6^b^	1⋅9	<0⋅0001	14⋅1^a^	1⋅3	4⋅8^b^	1⋅0	5⋅8^b^	1⋅7	<0⋅0001
Thiamin	0⋅1^a^	0⋅1	0⋅0^a^	0⋅0	0⋅0^a^	0⋅0	0⋅32	0⋅1^a^	0⋅0	0⋅0^a^	0⋅0	0⋅0^a^	0⋅0	0⋅21
Riboflavin	13⋅4^a^	1⋅5	3⋅5^b^	1⋅0	1⋅7^b^	0⋅9	<0⋅0001	12⋅4^a^	1⋅1	4⋅6^b^	1⋅1	3⋅0^b^	1⋅4	<0⋅0001
Niacin	0⋅0	0⋅0	0⋅0	0⋅0	0⋅0	0⋅0	-	0⋅0	0⋅0	0⋅0	0⋅0	0⋅0	0⋅0	-
Folate	7⋅4^a^	1⋅1	2⋅7^b^	0⋅8	0⋅6^b^	0⋅6	<0⋅0001	6⋅8^a^	0⋅8	1⋅9^b^	0⋅7	1⋅7^b^	1⋅0	<0⋅0001
Vitamin B6	0⋅4^a^	0⋅2	0⋅2^a^	0⋅1	0⋅0^a^	0⋅0	0⋅07	0⋅1^a^	0⋅1	0⋅1^a^	0⋅1	0⋅2^a^	0⋅1	0⋅93
Vitamin B12	0⋅8^a^	0⋅3	0⋅6^a^	0⋅3	0⋅6^a^	0⋅6	0⋅90	2⋅1^a^	0⋅5	0⋅7^a^	0⋅5	2⋅2^a^	1⋅2	0⋅11
Vitamin C	1⋅3^a^	0⋅5	0⋅1^b^	0⋅0	0⋅0^b^	0⋅0	0⋅006	2⋅0^a^	0⋅4	0⋅1^b^	0⋅1	0⋅0^b^	0⋅0	<0⋅0001
Potassium	19⋅0^a^	1⋅7	7⋅7^b^	1⋅4	1⋅7^c^	0⋅9	<0⋅0001	23⋅0^a^	1⋅5	11⋅7^b^	1⋅6	7⋅1^b^	1⋅9	<0⋅0001
Calcium	11⋅7^a^	1⋅4	5⋅3^b^	1⋅2	2⋅0^b^	0⋅9	<0⋅0001	11⋅5^a^	1⋅1	2⋅6^b^	0⋅8	0⋅9^b^	0⋅8	<0⋅0001
Magnesium	27⋅1^a^	1⋅9	11⋅8^b^	1⋅7	6⋅5^b^	1⋅7	<0⋅0001	19⋅2^a^	1⋅4	4⋅6^b^	1⋅0	3⋅1^b^	1⋅4	<0⋅0001
Iron	20⋅9^a^	1⋅7	13⋅5^b^	1⋅7	6⋅6^c^	1⋅8	<0⋅0001	14⋅4^a^	1⋅2	9⋅4^b^	1⋅5	8⋅5^b^	1⋅9	0⋅006
Zinc	19⋅4^a^	1⋅7	12⋅8^b^	1⋅7	8⋅0^b^	1⋅9	<0⋅0001	9⋅7^a^	1⋅0	3⋅2^b^	1⋅0	1⋅5^b^	0⋅9	<0⋅0001
Iodine	18⋅5^a^	1⋅6	7⋅9^b^	1⋅4	3⋅0^c^	1⋅1	<0⋅0001	15⋅4^a^	1⋅3	4⋅3^b^	1⋅0	3⋅7^b^	1⋅4	<0⋅0001
Selenium	21⋅5^a^	1⋅7	10⋅1^b^	1⋅5	5⋅2^b^	1⋅5	<0⋅0001	42⋅3^a^	1⋅7	34⋅7^b^	2⋅5	32⋅1^b^	3⋅4	0⋅005

* *P* values ≤0⋅003 were considered to be statistically significant to account for comparisons of fifteen outcomes. Different letters in the same row within the data panel for children or adults indicate a significant difference from Tukey's *post hoc* comparison.

### Percent contribution of yogurt to daily energy and nutrient intake in yogurt eaters

**Fig. 1.**
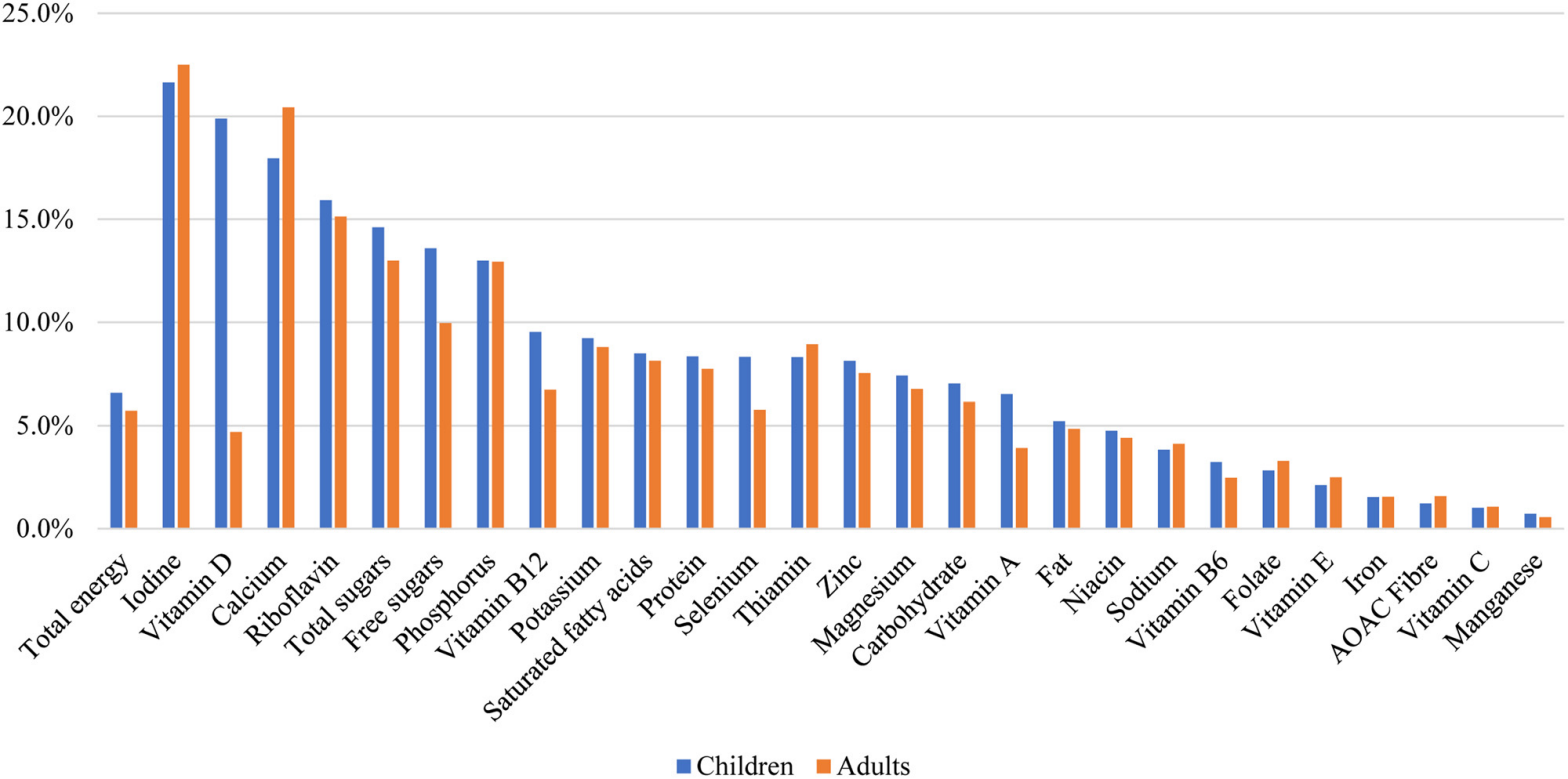
Percent contribution of yogurt to daily energy and nutrient intake in children and adults who were yogurt consumers in the United Kingdom, National Diet and Nutrition Survey 2014/15 to 2016/17.

Fig. 1 presents the percent contribution of yogurt to daily energy and nutrient intake in child and adult yogurt eaters, based on intake data from days when yogurt was consumed. Yogurt contributed to 6–7 % of calories in yogurt eaters in the UK. Notably, yogurt contributed to 22–23 % of iodine intake, 18–20 % of calcium intake, 15–16 % of riboflavin intake and about 13 % of phosphorus intake in yogurt eaters in children and adults. Yogurt also contributed to 19⋅9 % of vitamin D intake in children, although its contribution to vitamin D intake in adults was only 4⋅7 %. Yogurt contributed to approximately 13–15 % of total sugar and 10–14 % of free sugar in children and adults who consumed yogurt.

### Diet quality

**Fig. 2.**
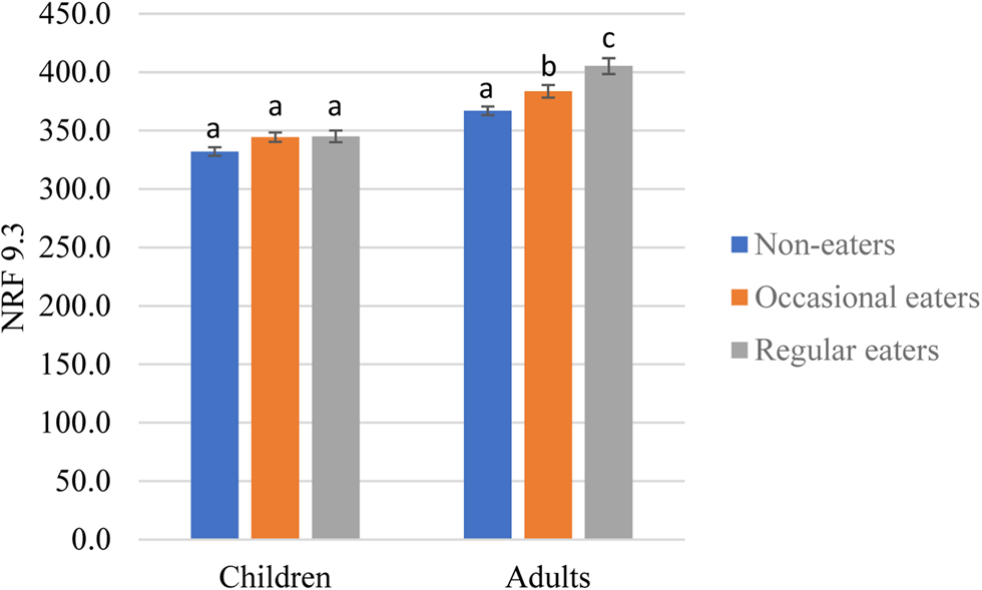
Adjusted diet quality as assessed by Nutrient Rich Food Index 9.3 (NRF 9.3) by frequency of yogurt consumption in children and adults in the United Kingdom, National Diet and Nutrition Survey 2014/15 to 2016/17. *Different letters within the same data panel for children or adults indicate a significant difference from Tukey's *post hoc* comparisons; results adjusted for age, gender and equivalised household income tertiles.

Significant positive associations between frequency of yogurt consumption and diet quality as assessed by adjusted NRF 9.3 scores were observed in both children (*P* = 0⋅045) and adults (*P* < 0⋅001). *Post hoc* comparisons also revealed that adult regular yogurt eaters had better diet quality than occasional eaters (*P* = 0⋅028) and non-eaters (*P* < 0⋅0001), and occasional eaters had better diet quality than non-eaters (*P* = 0⋅029) (Fig. 2).

## Discussion

The study reported associations between frequency of yogurt consumption and various dietary outcomes in both British children and adults using the most recent NDNS data. Yogurt is a popular food in the UK with approximately half of children and 40 % of adults consuming yogurt occasionally or regularly. In both children and adults, frequency of yogurt consumption was positively associated with higher intake of many vitamins and minerals whose intake should be encouraged, but not significantly associated with intake of free sugar and saturated fat. Yogurt consumers were more likely to meet or exceed dietary reference intakes of several nutrients.

While yogurt consumption was found to be more prevalent in children than that in adults, results from the present study supports previous findings that British children who consumed yogurt had higher intake of vitamins and minerals to encourage than those who did not consume yogurt^(19)^, and it confirms that similar differences could be found in the adult population, like studies of children and adults in United States^(12,13)^ or Canada^(15)^. The prevalence of yogurt consumption differed among these studies though, for example, 33 % of children aged 2–18 years in the US were frequent yogurt consumers^(13)^ whereas only 20 % of Canadians consumed yogurt on a given day^(15)^. Country-specific dietary tradition may partly explain for the differences, and methods used to estimate yogurt consumers in these studies may be another reason. For example, results from one single 24-h dietary recall may underestimate the prevalence of habitual yogurt consumption compare to studies that used multiple dietary recalls or food frequency questionnaires.

Results on associations between yogurt consumption and diet quality in this study support previous studies that yogurt consumers had a healthier diet than non-consumers^(12,13,15–17,19)^. The improvement in diet quality observed in this study may not be solely attributable to the inclusion of yogurt in their diet; it may also be due to higher intake of other healthy foods such as fruits and vegetables, as shown by results on intake of these food groups in this study. Several diet quality measurements, such as the Health Eating Index 2015^(36)^ and the Mediterranean diet score^(37)^, have included components like dairy, fruits and vegetables in their scoring algorithm; as such, consumption of a higher amount of fruits, vegetables or dairy products in yogurt consumers is expected to have a positive impact on their diet quality.

The present study identified that yogurt contributed to several key vitamins and minerals such as calcium and riboflavin. It was also noted that yogurt was a leading contributor to vitamin D intake in children but to a lesser extent in adults. This may be due to fortification of vitamin D in most children's yogurt products^(18)^. Food fortification is an effective strategy for addressing low intake of certain nutrients. In the present study, it was found that fortification of yogurt with vitamin D is making an important contribution to intake of this nutrient in the diets of children. As 16 % of adults in the UK had vitamin D deficiency (defined as serum 25-hydrooxy-vitamin D less than 25 nmol/l)^(38)^, vitamin D fortification in adult yogurt products may have public health implications.

Following the recommendation from the Scientific Advisory Committee on Nutrition, the NDNS replaced non-milk extrinsic sugar by free sugar in its dietary data since the 2014/15 data release, which by definition, included all monosaccharides and disaccharides added to food, as well as sugars naturally present in honey, syrups, fruit and vegetables juice, purees or pastes^(22)^. The availability of free sugar data in the NDNS since the release of the 2014/15 data now makes it feasible to explore associations between yogurt consumption and free sugar intake in a nationally representative sample in the UK, for the first time, and it was found that there was no significant association between frequency of yogurt consumption and intake of free sugar in both children and adults in the UK. Intake of total sugar, however, is higher in yogurt eaters. Taken together, this suggests naturally occurring sugars in the diets from yogurt eaters are accounting for their higher intake of total sugar.

It was found that frequency of yogurt consumption was positively associated with nutrient adequacy for many vitamins and minerals in both children and adults in the UK. Similar results in children were reported in a previous study^(19)^ and results in adults were reported for the first time, to our knowledge, which revealed a similar pattern. Certain nutrients, such as calcium, iron, potassium and selenium, warrants public health attention as the prevalence of intake below LRNI was not low, particularly in those who did not eat yogurt.

The study has several limitations. First, the study used cross-sectional data and causal inference should not be made. Second, similar to most epidemiological studies of yogurt consumption^(12–16,19)^, the study did not examine the type of yogurt consumed due to smaller sample sizes. Future studies that compare different types of yogurt, such as regular yogurt, Greek yogurt and probiotic yogurt, are warranted. Lastly, the study has limited its scope of analyses to dietary outcomes without investigation of associations of yogurt consumption with health outcomes; prospective cohort studies and randomised controlled trials may be better approaches to address those questions. Despite these limitations, the study used the most recent data from a nationally representative sample in the UK, and investigated associations between frequency of yogurt consumption and dietary outcomes, building on previous studies that compared yogurt consumers to non-consumers based on one-day dietary data when determination of frequency of consumption was not feasible^(14,15)^.

In conclusion, frequent yogurt consumption was positively associated with dietary intake of key vitamins and minerals, nutrient adequacy and diet quality in the UK, based on the most recent NDNS data. People who ate yogurt also had higher intake of certain food groups that are encouraged by the UK Eatwell Guide, while no difference in free sugar intake was found compared to those who did not consume yogurt. Policy makers should consider recommendations to consume yogurt as a potential strategy to increase consumption of under-consumed nutrients.
